# Hybrid dielectrics composed of Al_2_O_3_ and phosphonic acid self-assembled monolayers for performance improvement in low voltage organic field effect transistors

**DOI:** 10.1186/s40580-018-0152-3

**Published:** 2018-07-25

**Authors:** Sukjae Jang, Dabin Son, Sunbin Hwang, Minji Kang, Seoung-Ki Lee, Dae-Young Jeon, Sukang Bae, Sang Hyun Lee, Dong Su Lee, Tae-Wook Kim

**Affiliations:** Functional Composite Materials Research Center, Institute of Advanced Composite Materials, Korea Institute of Science and Technology, Wanju-gun, Jeollabuk-do 55324 Republic of Korea

**Keywords:** Hybrid dielectrics, ALD Al_2_O_3_, PA-SAM, Phosphonic acid, Water contact angle, Organic transistor

## Abstract

Low voltage operational organic transistors (< 4 V) based on pentacene were successfully fabricated with hybrid dielectric films composed of aluminum oxide using atomic layer deposition and various phosphonic acid-based self-assembled monolayers as the gate dielectrics. High capacitances up to 279 nF/cm^2^, low leakage current densities of 10^−8^ A/cm^2^ at 6 V, and high breakdown fields up to 7.5 MV/cm were obtained. The transistors with the octadecylphosphonic acid hybrid dielectric exhibited an improved saturation mobility of 0.58 cm^2^/Vs, a subthreshold slope of 151 mV/decade, a threshold voltage of − 1.84 V and an on–off current ratio of 10^6^. The low surface energies of the self-assembled monolayers having non-polar terminal groups, such as methyl and pentafluorophenoxy, improved the carrier conduction of the transistors due to the pentacene growth with an edge-on orientation for low voltage operation. The pentafluorophenoxy end-group showed an accumulation of holes at the semiconductor-dielectric interface.

**Electronic supplementary material:**

The online version of this article (10.1186/s40580-018-0152-3) contains supplementary material, which is available to authorized users.

Organic field-effect transistors (OFETs) have great potential for a wide variety of applications, such as flexible displays, electronic paper, radiofrequency identification tags and sensors, due to their low cost, low temperature fabrication, solution process compatibility, light weight and mechanical flexibility compared with conventional inorganic materials [1–4]. The relative high operating voltages of OFETs have hindered their development in the commercial market. To implement low voltage operating OFETs, significant efforts have been devoted to increasing the capacitive density of gate dielectrics by decreasing the thickness of the gate dielectrics or increasing the dielectric constant (*k*) of the dielectric films [5, 6].

High-*k* dielectric films can induce greater surface charge densities at the semiconductor-dielectric interface than those of low-*k* gate dielectrics [7]. High-*k* dielectric films, such as metal oxides, were typically deposited using atomic layer deposition (ALD) based on the sequential use of a self-limited surface reaction [8, 9]. The ALD technique has various advantages, such as precise thickness control, high quality films based on layer-by-layer growth, a lower growth temperature (< 350 °C) than that of classical chemical vapor deposition methods, a high uniformity, an excellent conformality over high aspect ratio structures, and dense and pinhole free films [8, 10]. Among the various high-*k* dielectrics, aluminum oxide (Al_2_O_3_) is considered as a potential gate dielectric film of field effect transistors due to its thermodynamic stability on Si up to high temperatures, mechanical robustness and highly insulating properties that are due to its high band gap (8.7 eV) and medium-*k* value (8–9) [7].

Another strategy for low voltage OFETs is improving the charge transport in organic semiconductors. Charge transport occurs in the few monolayers near the interface between the organic semiconductor/dielectric and is limited by the thermally activated hopping process between the molecules in the disordered regions [11, 12]. To improve the device performance, the disordered region of the semiconductor should be suppressed, and additional interface treatments are required for optimal charge transport. For example, the molecular parameters of organic semiconductors were modified by controlling their regioregularity, molecular weight, side chain length, doping level and end-group. Interface engineering has been introduced to improve the molecular ordering, orientation, assembly, packing and film morphology of organic semiconductors [13]. Self-assembled monolayers (SAMs) are good candidates for effective surface treatment due to their high-packing density, being only a few nm thick and allowing interface control with end-functional groups [14]. Among various SAMs with different binding groups, such as carboxyl groups, thiols and silanes, phosphonic acid self-assembled monolayers (PA-SAMs) have attracted attention due to readily assembling on activated metal oxides because their reaction is not limited by the contents of surface hydroxyl groups and supplies their own hydroxyl moieties [15]. They also have the advantages of stability and prevention of homocondensation between phosphonic acid molecules compared with other binding groups, resulting in the highly ordered, dense and robust monolayers of phosphonic acid molecules on metal oxide surfaces.

Therefore, hybrid dielectrics composed of PA-SAMs on a high-*k* dielectric are excellent candidates for OFET applications [15]. They are suitable for gate dielectrics in OFETs due to their low voltage operation, low gate leakage currents and interface modification by the functional end-groups of SAMs. In this letter, we introduce various hybrid dielectrics comprised of PA-SAMs on ALD Al_2_O_3_ and further investigate the relationship between various functional end-groups of PA-SAMs and the device performances of OFETs.

Figure 1 illustrates the schematic structure of an OFET with a hybrid dielectric consisting of ALD Al_2_O_3_ and PA-SAMs and molecular structures of various PA-SAMs with different functional end-groups. We prepared seven kinds of PA-SAMs to investigate the effects of the alkyl chain length and functional end-groups on the OFET performance. Hexylphosphonic acid (HPA), dodecylphosphonic acid (DDPA), octadecylphosphonic acid (ODPA), phosphonohexadecanoic acid (PHDA), 12-mercaptododecylphosphonic acid (MDPA), 12-pentafluorophenoxydodecylphosphonic acid (PFPA), 11-hydroxyundecylphosphonic acid (HUPA), pentacene (99.9% purity) and isopropanol (99.8% purity) were purchased commercially from Sigma-Aldrich, Korea and used as received. HPA (C6), DDPA (C12) and ODPA (C18) are methyl-terminated PA-SAMs with increasing alkyl chain lengths ranging from C6 to C18. PHDA, MDPA, PFPA and HUPA have different end functionalized terminals with the carboxyl group, thiol, (2,3,4,5,6-pentafluorophenoxy) group and hydroxyl group, respectively (Fig. 1b).

**Fig. 1:**
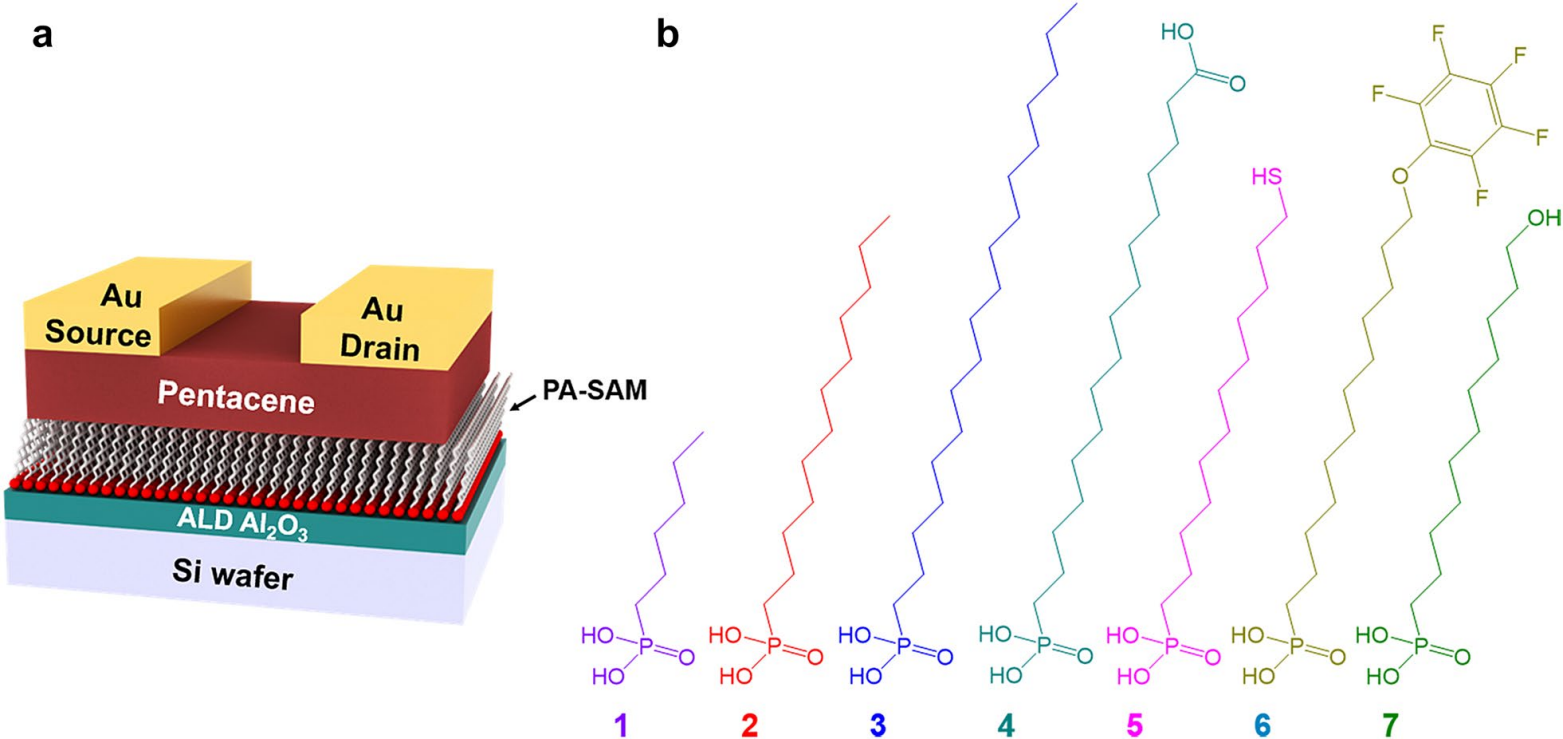
**a** Schematic structure of an OFET with a hybrid dielectric. **b** Molecular structures of the various PA-SAMs used in this study. [1: Hexylphosphonic acid (HPA) (C6), 2: dodecylphosphonic acid (DDPA) (C12), 3: octadecylphosphonic acid (ODPA) (C18), 4: 16-phosphonohexadecanoic acid (PHDA), 5: 12-mercaptododecylphosphonic acid (MDPA), 6: 12-pentafluorophenoxydodecylphosphonic acid (PFPA), 7: 11-hydroxyundecylphosphonic acid (HUPA)]

Heavily n-doped bare Si (100) substrates were diced, cleaned using ultra-sonication in acetone and isopropanol for 10 min, and then dried with nitrogen gas. An *n*^+^-doped bare Si wafer acted as the back-gate electrode in the OFETs. The cleaned substrates were annealed in a convection oven for 1 h to remove residual solvent. Al_2_O_3_ dielectric films were deposited on the cleaned substrates by 200 ALD cycles (Lucida D100 ALD, NCD) at a process temperature of 200 °C. Trimethylaluminum (TMA, Al(CH_3_)_3_) and water were used as the Al and O precursors, respectively. The growth rate per cycle (GPC) was calculated to be ~ 0.12 nm/cycle.

Phosphonic acid solutions were prepared by dissolving 3 mM of each phosphonic acid in 1 ml of isopropanol. The Al_2_O_3_ thin films were exposed to UV-Ozone for 30 min to generate enough density of the hydroxyl groups on the surface for the SAM treatment. The prepared PA-SAM solutions after filtration with a 0.45 µm PTFE membrane were immediately spin-coated on the UV-Ozone treated Al_2_O_3_ thin films at a spin rate of 3000 rpm for 20 s and annealed at 140 °C for 10 min in a nitrogen-filled glovebox. The annealed substrates were rinsed using ultra-sonication in isopropanol for 10 min to remove any remaining phosphonic acids except for the self-assembled monolayer on the surface.

Figure 2a displays images of water droplets on the hybrid dielectrics that were modified with various PA-SAM molecules and the measured water contact angles. The contact angle of the reference Al_2_O_3_ surface was measured to be less than 10°, implying a hydrophilic property. After the formation of PA-SAM molecules on the Al_2_O_3_ dielectric, the water contact angles were dramatically changed depending on the kind of molecules that were formed. Alkyl-phosphate SAMs and PFPA exhibited a low energy surface with a high contact angle (> 100°) due to their methyl and fluorinated functional groups, respectively [15]. The contact angle differences between the PA-SAMs were explained by the polarity of the end-functional groups of the SAM molecules. Non-polar end-groups, such as methyl and 2,3,4,5,6-pentafluorophenoxy of alkyl-phosphate SAMs and PFPA, enabled highly hydrophobic surfaces based on the their remarkably low surface energies compared to other SAMs. However, MDPA, HUPA and PHDA exhibited relatively lower water contact angles due to their increased polarity of the end-groups, such as the thiol, hydroxyl and carboxyl groups. Alkyl-phosphate SAMs (HPA, DDPA and ODPA) have slightly increased contact angles as the alkyl chain length is increased. These phenomena were attributed to the fact that increasing an alkyl chain length increases the Van der Waals interaction between the alkyl chains, leading to a more crystalline, all-*trans* conformation of the chains and a highly packed hydrophobic monolayer with lower surface energies [16, 17].

**Fig. 2:**
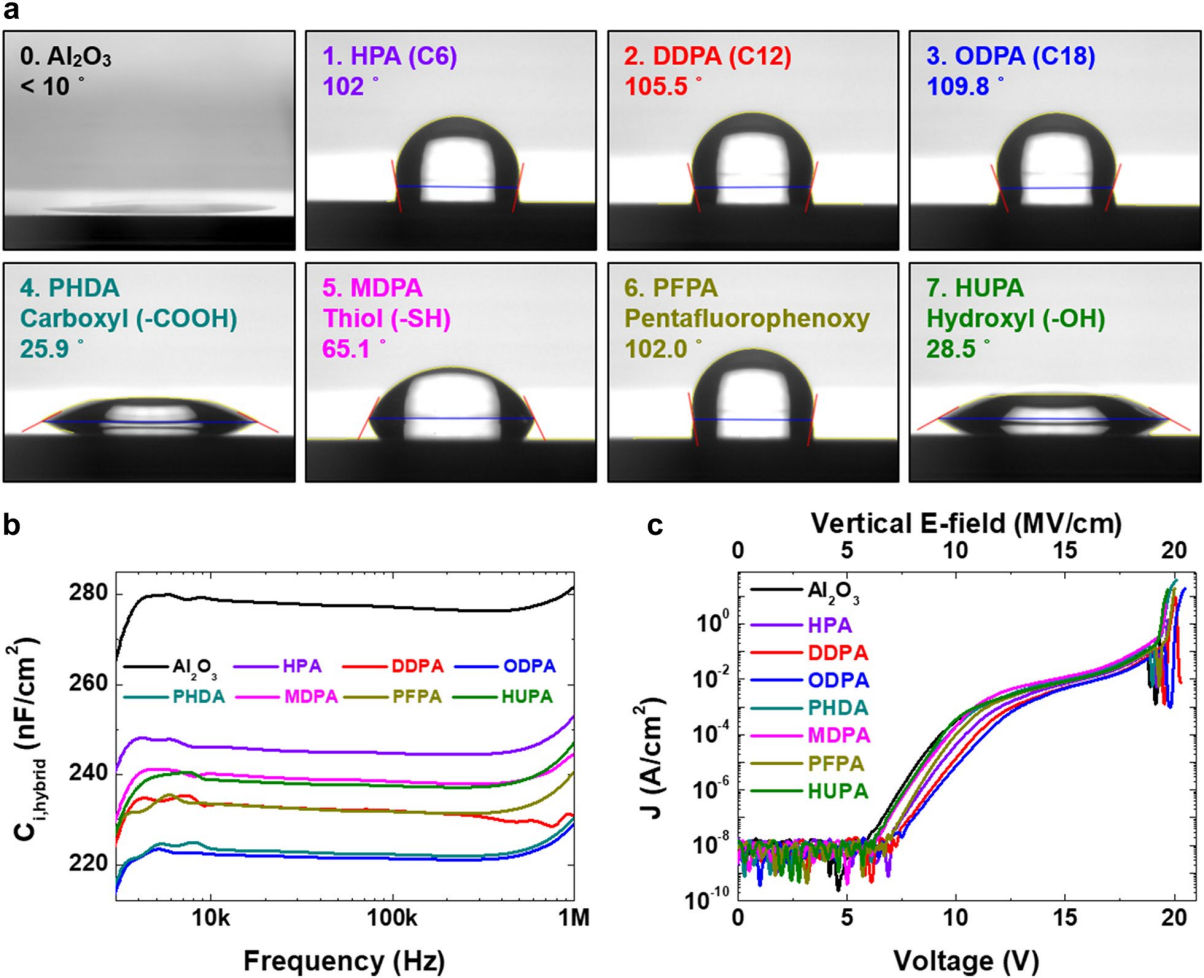
**a** Water droplets on hybrid dielectrics and measured contact angles, **b** capacitive densities–frequency (*C*_*i*_–*F*) and **c** leakage current densities–voltage (*J–V*) of hybrid dielectrics with various PA-SAMs

To investigate the electrical properties of hybrid dielectrics with diverse PA-SAMs, metal–insulator-semiconductor (MIS) capacitors and OFETs were both prepared on the same substrates. Pentacene films (50 nm thick) were evaporated onto a hybrid dielectric using a shadow mask with a deposition rate of 0.2–0.3 Å/s at 10^−7^ Torr. Gold (50 nm thick) source/drain electrodes (250 μm × 200 μm) for the OFET and top contact electrodes (square, 200 μm × 200 μm) for the MIS capacitors were concurrently deposited using thermal evaporation at 10^−6^ Torr. The electrical characteristics of the hybrid dielectric were measured using a probe station, a semiconductor parameter analyzer (4145B, HP) and a semiconductor characterization system (4200-SCS, Keithley) in a nitrogen-filled glove box system. Additional file 1: Figure S1a displays the optical microscopic image of the OFET and MIS capacitor used for the characterization of the hybrid dielectric.

The capacitance measurements and breakdown measurements were evaluated via MIS capacitors. Figure 2b displays the measured capacitive densities (*C*_*i*_) of hybrid dielectrics as a function of frequencies from 300 Hz to 1 MHz. We took the *C*_*i*_ values at 10 kHz for the convenient characterization. *C*_*i*_ of the reference Al_2_O_3_ without a PA-SAM was measured to be ~ 279 nF/cm^2^. The dielectric constant *k* can be calculated from the following equation:

1 $${{\text{C}}_i} = {\varepsilon_0}\frac{k}{t_i}$$

where *ε*_*0*_ is the permittivity of free space and *t*_*i*_ is the thickness of the insulator. The thickness of Al_2_O_3_ produced by 200 ALD cycles was measured to be ~ 23 nm, and the calculated *k* of the reference Al_2_O_3_ was ~ 7.2, which is lower than the ideal value of bulk Al_2_O_3_ (*k* ~ 8–9) [18] but higher than the typical value of sputtering-grown Al_2_O_3_ (*k* ~ 7) [19]. The discrepancy of the ideal and measured value of the *k* of Al_2_O_3_ might originate from the amorphous phase, the existence of native SiO_2_, the series capacitance, the depletion of the n^+^ Si bottom electrode or an interface charge.

*C*_*i*_ is formed by the two-series capacitive densities and is described as follows:

2 $$\frac{1}{C_i} = \frac{1}{{{C_{i,A{l_2}{O_3}}}}} + \frac{1}{{{C_{i,PA - SAM}}}}$$

where ${C_{i,A{l_2}{O_3}}}$ and *C*_*i,PA*-*SAM*_ are the capacitive densities of the Al_2_O_3_ dielectric and the PA-SAM, respectively. From Eqs. (1) and (2), the *C*_*i,PA*-*SAM*_ and *k* of each PA-SAM, respectively, were extracted and summarized with related parameters in Table 1.

**Table 1:** Thin film parameters of the hybrid dielectrics with PA-SAMs

Dielectric	Molecular length^a^ (nm)	*t*_*i*_^b^ (nm)	Contact angle (º)	*C*_*i*_ at 10 kHz (nF/cm^2^)	Estimated *k*^c^
Al_2_O_3_	–	23	< 10	279	7.2
HPA	1	0.91	102	246	2.2
DDPA	1.74	1.59	105.5	230	2.6
ODPA	2.49	2.27	109.8	222	2.8
PHDA	2.25	2.05	25.9	223	2.6
MDPA	1.9	1.74	65.1	240	3.4
PFPA	2.24	2.04	102	234	3.3
HUPA	1.72	1.57	28.5	239	3.0

Molecular length (nm), thickness of insulator (*t*_*i*_) (nm), contact angle (°), capacitive density (*C*_*i*_) at 10 kHz (nF/cm^2^) and Estimated dielectric constant *k*

^a^ The molecular lengths of the PA-SAMs were estimated by assuming the conditions, such as the ideal bonding length and flat molecules according to the alkyl chain axis. The molecular lengths were defined as the distance between the hydrogen atom at the phosphonic acid and the opposite end atom along the alkyl chain axis

^b^ *t*_*i*_ was the thickness of each Al_2_O_3_ and PA-SAM layer (not hybrid dielectric). The *t*_*i*_ of the reference Al_2_O_3_ was a measured value. The *t*_*i*_ of the PA-SAMs were calculated by assuming the calculated molecular lengths, the tilted bonding angle to the substrate (24°) and full coverage with a high density on the substrate

^c^ *k* was the calculated dielectric constant of each Al_2_O_3_ and PA-SAM layer (not hybrid dielectric) using Eqs. (1) and (2)

The overall capacitive densities of the hybrid dielectrics (*C*_*i*_) were decreased from 279 nF/cm^2^ for the reference Al_2_O_3_ to 222–245 nF/cm^2^ for each PA-SAM treatment. These results provide evidence of the molecular layer formation on the Al_2_O_3_ surface, which contributed to reducing their capacitive densities. The *C*_*i*_ of the hybrid dielectrics are sufficient for an operating voltage of less than 5 V but can be optimized by further reducing the thickness of the Al_2_O_3_ for lower voltage operation of the OFETs.

Figure 2c shows the leakage current densities as a function of the applied voltage to the MIS capacitor. The breakdown electric fields were extracted and ranged from 7.5 to 8 MV/cm based on the PA-SAM type, which was better than the Al_2_O_3_ deposited by RF-magnetron sputtering system (3–5 MV/cm) [19] but smaller than that of thermally grown SiO_2_ (14 MV/cm) [20]. The leakage current densities of 10^−8^ A/cm^2^ were maintained at 6 V without the appearance of tunneling currents. The high capacitive densities (*C*_*i*_ > 220 nF/cm^2^) and low leakage current densities (10^−8^ A/cm^2^ at 6 V) of the hybrid dielectrics (*t*_*i*_ < 25 nm), which originated from the high-*k* and dense inorganic thin film of Al_2_O_3_, were adequate for low voltage operation of the OFETs.

For the sake of the relationship between the hybrid dielectric with various PA-SAMs and the electrical performance of the OFETs, the OFETs (Channel width *W* = 200 μm and channel length *L* = 15 μm) were fabricated in a bottom gate, top contact architecture on Al_2_O_3_/PA-SAM hybrid dielectrics. Figure 3 shows the transfer characteristics and the square root of I_DS_ of the OFETs with various PA-SAMs as a function of *V*_*GS*_ at *V*_*DS*_ = − 4 V in the saturation regime. The drain current of the OFET in the saturation regime is described as follows:

3 $${\left( {{I_{DS}}} \right)_{sat}} = {\mu_{sat}}{C_i}\frac{W}{L}{\left( {{V_{GS}} - {V_{Th}}} \right)^2}$$

where (*I*_*DS*_)_*sat*_ is the drain current in the saturation regime, *μ*_*sat*_ is the field effect mobility in the saturation regime, *V*_*Th*_ is the threshold voltage, *V*_*DS*_ is the source-drain voltage and *V*_*GS*_ is the gate-drain voltage. We could estimate the threshold voltage as the *x* intercept of the linear fit for the square root of *I*_*DS*_-*V*_*GS*_ and the saturation field effect mobility of OFETs using Eq. (3). The subthreshold slope (*SS*) was calculated using the inverse slope of log (*I*_*DS*_)-*V*_*GS*_ in the subthreshold region of the one-decade current increase.

**Fig. 3:**
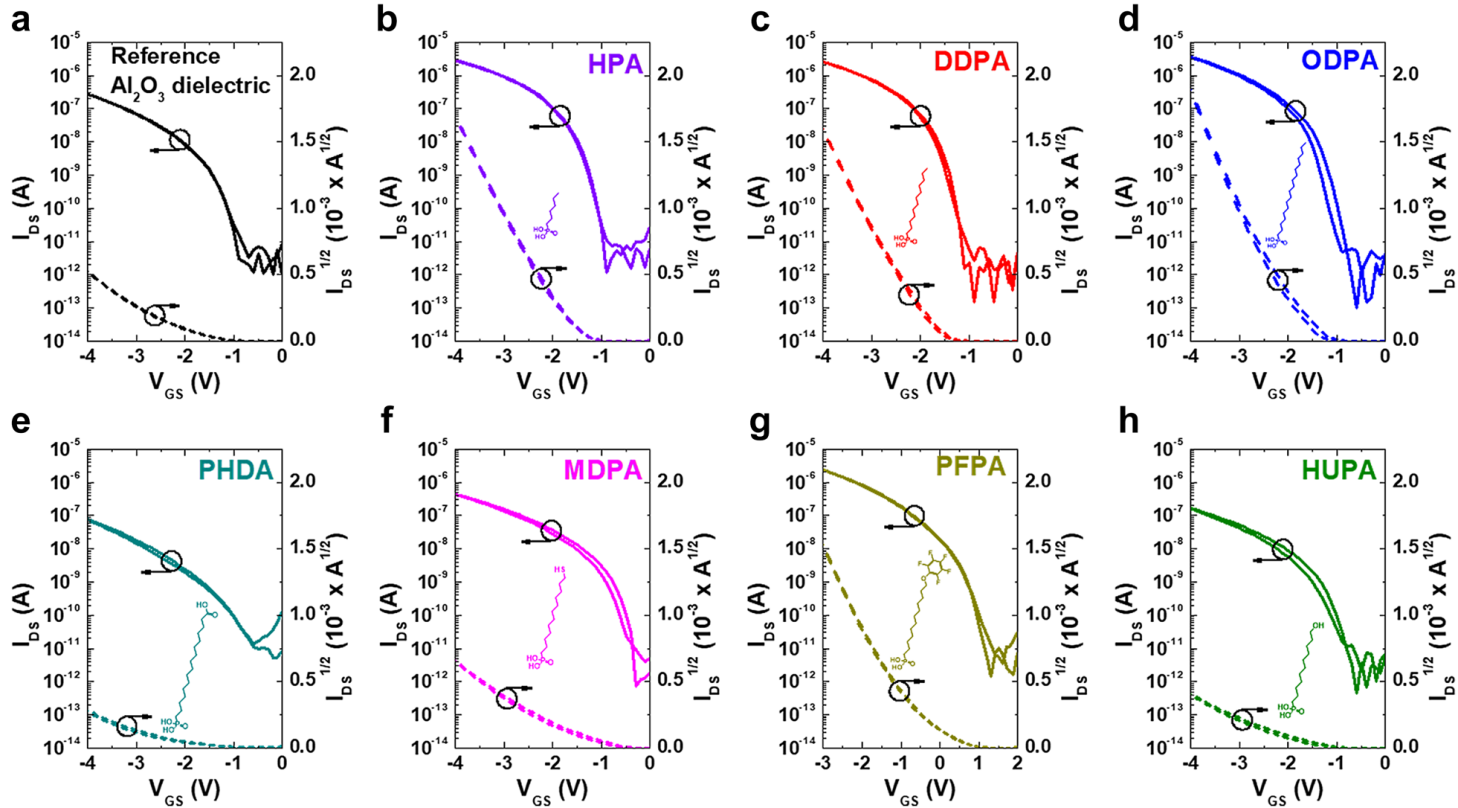
Transfer characteristics (solid line) and the square root of *I*_*DS*_ (dashed line) as a function of *V*_*GS*_ of the OFETs with hybrid dielectrics with various PA-SAMs **a** reference Al_2_O_3_, **b** HPA, **c** DDPA, **d** ODPA, **e** PHDA, **f** MDPA, **g** PFPA and **h** HUPA

For comparison, reference transistors with an Al_2_O_3_ dielectric (*t*_*i*_ = 23 nm, without PA-SAM) were prepared (Fig. 3a). The reference transistor showed stable *p*-type transfer characteristics and negligible hysteresis with an operating voltage below − 4 V. The reference transistor exhibited substandard performances, such as the field effect mobility in the saturation regime (*μ*_*sat*_), the subthreshold slope (*SS*), the threshold voltage (*V*_*Th*_) and the on–off current ratio (*I*_*on*_*/I*_*off*_), which were approximately 0.05 cm^2^/Vs, 345 mV/decade, − 2.18 V and ~ 10^5^, respectively. The output characteristics of the OFETs as a function of *V*_*GS*_ are shown in Additional file 1: Figure S2. The device characteristics of the OFETs with hybrid dielectrics of the PA-SAM type are summarized in Table 2.

**Table 2:** Device characteristics of OFETs with hybrid dielectrics

Dielectric	Contact angle (º)	*μ*_*sat*_ (cm^2^/Vs)	*SS* (mV/decade)	*V*_*Th*_ (V)	*I*_*on*_ */I*_*off*_
Al_2_O_3_	< 10	0.05	345	− 2.18	~ 10^5^
HPA	102	0.35	151	− 1.71	~ 10^6^
DDPA	105.5	0.41	173	− 1.83	~ 10^6^
ODPA	109.8	0.58	135	− 1.84	~ 10^6^
PHDA	25.9	0.02	493	− 2.35	~ 10^4^
MDPA	65.1	0.05	197	− 1.64	~ 10^5^
PFPA	102	0.27	198	− 0.58	~ 10^6^
HUPA	28.5	0.03	196	− 1.95	~ 10^5^

The contact angle (º), saturation field effect mobility *μ*_*sat*_ (cm^2^/Vs), subthreshold slope *SS* (mV/decade), threshold voltage *V*_*Th*_ (V), on–off current ratio *I*_*on*_*/I*_*off*_

*μ*_*sat*_ (cm^2^/Vs), *SS* (mV/decade), *V*_*Th*_ (V) and *I*_*on*_*/I*_*off*_ were calculated

The OFETs with a hybrid dielectric commonly exhibited transfer characteristics with negligible hysteresis with an operating voltage below − 4 V in the accumulation mode; the transfer characteristics were analogous to the reference transistor. The gate leakage currents (*I*_*GS*_) of the hybrid dielectrics were negligible compared with the drain currents during the operation (less than 4 V) as discussed above (Fig. 2c and Additional file 1: Figure S1). The OFETs with the alkyl-phosphate SAMs and PFPA exhibited noticeable improvements in the device performances, including one order of magnitude of the saturation field-effect mobility (*μ*_*sat*_) and the lower threshold voltage (*V*_*Th*_), as well as one order of magnitude of the on–off current ratio (*I*_*on*_*/I*_*off*_) compared with the reference transistor. The most important parameter of the OFET charge transport is the carrier mobility. The hybrid dielectric with ODPA showed the highest saturation mobility of 0.58 cm^2^/Vs, the lowest subthreshold slope of 151 mV/decade, a threshold voltage of − 1.84 V and an on–off current ratio of 10^6^. The all methyl-terminated alkyl-phosphate SAMs have common higher performances among the PA-SAMs because of their hydrophobic surface based on their low surface energy [15]. The formation of hydrophobic self-assembled monolayers (SAMs) on inorganic dielectric surfaces induces the edge-on orientation of the organic semiconductor on hydrophobic SAMs during the organic semiconductor growth [14]. In the horizontal OFET configuration, the in-plane π–π stacking of the edge-on orientation is highly suitable for high in-plane charge transport from the source to the drain, resulting in the improvement of the mobility of the OFET. Furthermore, the low surface energy of the hydrophobic surface promotes a greater diffusion of organic semiconductor molecules during the thin film growth and enables a larger grain size of the organic semiconductor [21, 22]. The large grains of pentacene suppress the disordered regions, limiting their charge transport by the thermally activated hopping process and resulting in an improvement of the mobility. PFPA also showed a high mobility of 0.27 cm^2^/Vs, which was comparable to the alkyl-phosphates of the SAMs due to their high hydrophobic surface, as well as the lowest *V*_*Th*_ of − 0.58 V. This threshold voltage shift of PFPA compared with the reference transistor originated from the charge carrier accumulation of holes at the semiconductor-dielectric interface by the electronegative fluorine atoms of the 2,3,4,5,6-pentafluorophenoxy end-functional group [23–25]. PHDA exhibited the lowest *V*_*Th*_ of − 2.35 V. The relative hydrophilic PA-SAMs with a high surface energy tended to induce a growth of the pentacene semiconductor with a face-on orientation due to the stronger substrate–film interactions and represented the lower mobilities of the OFETs [22].

Figure 4 shows the saturation mobilities and subthreshold slopes of the OFETs as a function of the water contact angle. This figure again presents how the dependence of the device performances significantly depends on the type of PA-SAMs. The non-polar end-functional groups of PA-SAMs with a lower the surface energy, resulted in pentacene with the edge-on orientation and a large grain. Effective in-plane charge carrier transport occurs due to the π–π stacking of pentacene with the edge-on orientation and suppressed disordered regions with larger sized grains [26, 27]. PFPA with the pentafluorophenoxy end-group showed good device performances comparable to PA-SAMs with the methyl terminal group due to the low surface energy and the accumulation of holes, which are majority carriers in the pentacene semiconductor, at the semiconductor-dielectric interface caused by fluorinated moieties. These results demonstrate that a hybrid dielectric based on a hydrophobic surface provides a powerful method to improve the device performance of OFETs.

**Fig. 4:**
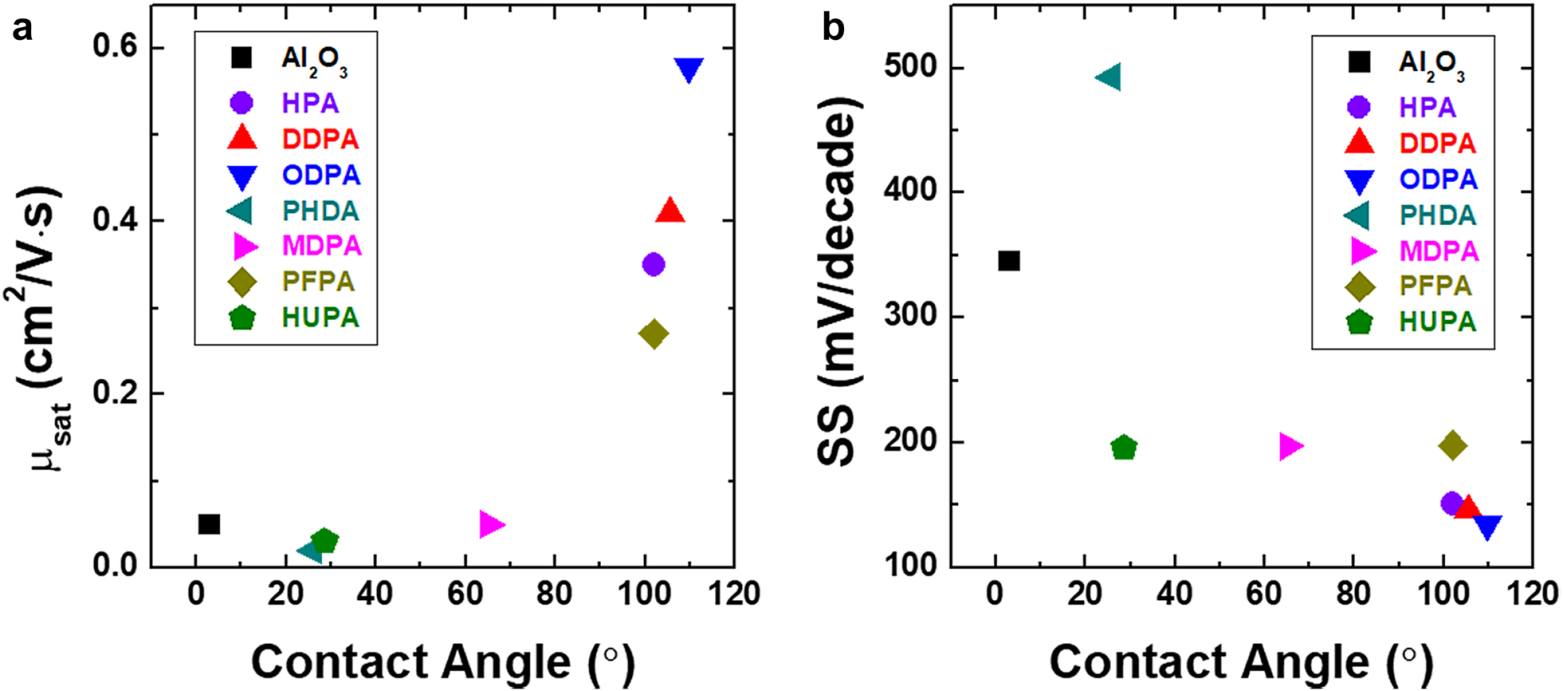
**a** Saturation field-effect mobilities and **b** subthreshold slopes of hybrid dielectrics with various PA-SAMs as a function of the water contact angle

In summary, low voltage operational OFETs below 4 V were successfully fabricated with hybrid dielectric films. Hybrid dielectric films were prepared using ALD and the consecutive assembly of various PA-SAMs using simple spin-coating. The water contact angles, capacitive densities and leakage current densities were measured for the characterization of the hybrid dielectric films. The methyl terminated alkyl-phosphates and PFPA have hydrophobic surfaces with a low surface energy due to their non-polar end-functional groups. The dielectric constant *k* of the Al_2_O_3_ and PA-SAM layers were extracted. The OFETs were fabricated for the evaluation of the hybrid dielectrics for transistor applications. The OFET with ODPA exhibited the highest saturation mobility of 0.58 cm^2^/Vs among the PA-SAMs. The edge-on orientation of pentacene, which was promoted by the lower surface energies of PA-SAM due to the non-polar end-groups, improved the carrier conduction between the source-drain electrodes compared to bare Al_2_O_3_. PFPA showed a positive *V*_*Th*_ shift due to the accumulation of holes at the semiconductor-dielectric interface. This hybrid dielectric platform can be compatible with low voltage transistor applications depending on the polarity of the solution processed and the vacuum deposited organic semiconductor.

## Additional file

**Additional file 1: Figure S1.** (a) Optical microscopic image of our OFET and MIS capacitor with a hybrid dielectric. (b) Gate leakage currents as a function of the gate voltage (*I*_*GS*_-*V*_*GS*_) of the OFETs that include hybrid gate dielectrics with various PA-SAMs. **Figure S2.** Output characteristics as a function of the gate voltage (*I*_*DS*_-*V*_*GS*_) of the OFETs with hybrid dielectrics with various PA-SAMs (a) reference Al_2_O_3_ (b) HPA, (c) DDPA (d) ODPA, (e) PHDA, (f) MDPA, (g) PFPA and (h) HUPA.

## Abbreviations

OFETs: organic field effect transistors
*k*: dielectric constant
ALD: atomic layer deposition
Al_2_O_3_: alumium oxide
SAM: self-assembled monolayer
PA-SAM: phosphonic acid self-assembled monolayer
HPA: hexylphosphonic acid
DDPA: dodecylphosphonic acid
ODPA: octadecylphosphonic acid
PHDA: phosphonohexadecanoic acid
MDPA: 12-mercaptododecylphosphonic acid
PFPA: 12-pentafluorophenoxydodecylphosphonic acid
HUPA: 11-hydroxyundecylphosphonic acid
MIS: metal–insulator-semiconductor
*C*_*i*_: capacitive density
*t*_*i*_: thickness of insulator
*I*_*DS*_: the drain current
(*I*_*DS*_)_*sat*_: the drain current in saturation regime
*μ*_*sat*_: the field effect mobility in saturation regime
*V*_*Th*_: the threshold voltage
*V*_*DS*_: the source-drain voltage
*V*_*GS*_: the gate-drain voltage
*SS*: substhreshold slope
*I*_*on*_*/I*_*off*_: on–off current ratio
