# Supplementary material for: Hybrid dielectrics composed of Al2O3 and phosphonic acid self-assembled monolayers for performance improvement in low voltage organic field effect transistors
Source: Nano Converg. 2018 Jul 25;5:20. doi: 10.1186/s40580-018-0152-3 (PMC6061253; doi:10.1186/s40580-018-0152-3)
Supplement: Supplementary file 1 — Additional file 1: Figure S1. (a) Optical microscopic image of our OFET and MIS capacitor with a hybrid dielectric. (b) Gate leakage currents as a function of the gate voltage (IGS-VGS) of the OFETs that include hybrid gate dielectrics with various PA-SAMs. Figure S2. Output characteristics as a function of the gate voltage (IDS-VGS) of the OFETs with hybrid dielectrics with various PA-SAMs (a) reference Al2O3 (b) HPA, (c) DDPA (d) ODPA, (e) PHDA, (f) MDPA, (g) PFPA and (h) HUPA. [file 40580_2018_152_MOESM1_ESM.docx]

Additional file for

**Hybrid dielectrics composed of Al_2_O_3_ and phosphonic acid self-assembled monolayers for performance improvement in low voltage organic field effect transistors**

Sukjae Jang, Dabin Son, Sunbin Hwang, Minji Kang, Seoung-Ki Lee, Dae-Young Jeon, Sang Hyun Lee, Dong Su Lee and Tae-Wook Kim^*^

^*^ Corresponding author. Email: [twkim@kist.re.kr](mailto:twkim@kist.re.kr)

**
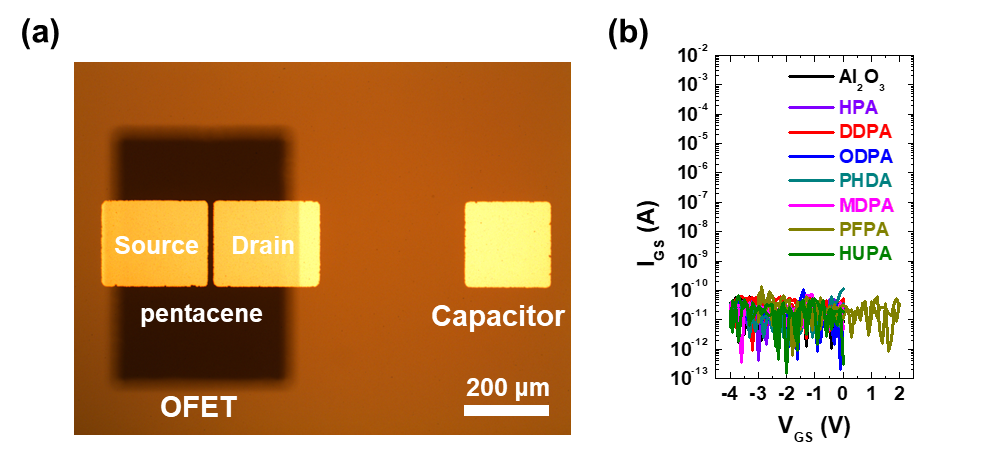
**

**Figure S1.** (a) Optical microscopic image of our OFET and MIS capacitor with a hybrid dielectric. (b) Gate leakage currents as a function of the gate voltage (*I_GS_*-*V_GS_*) of the OFETs that include hybrid gate dielectrics with various PA-SAMs.

**
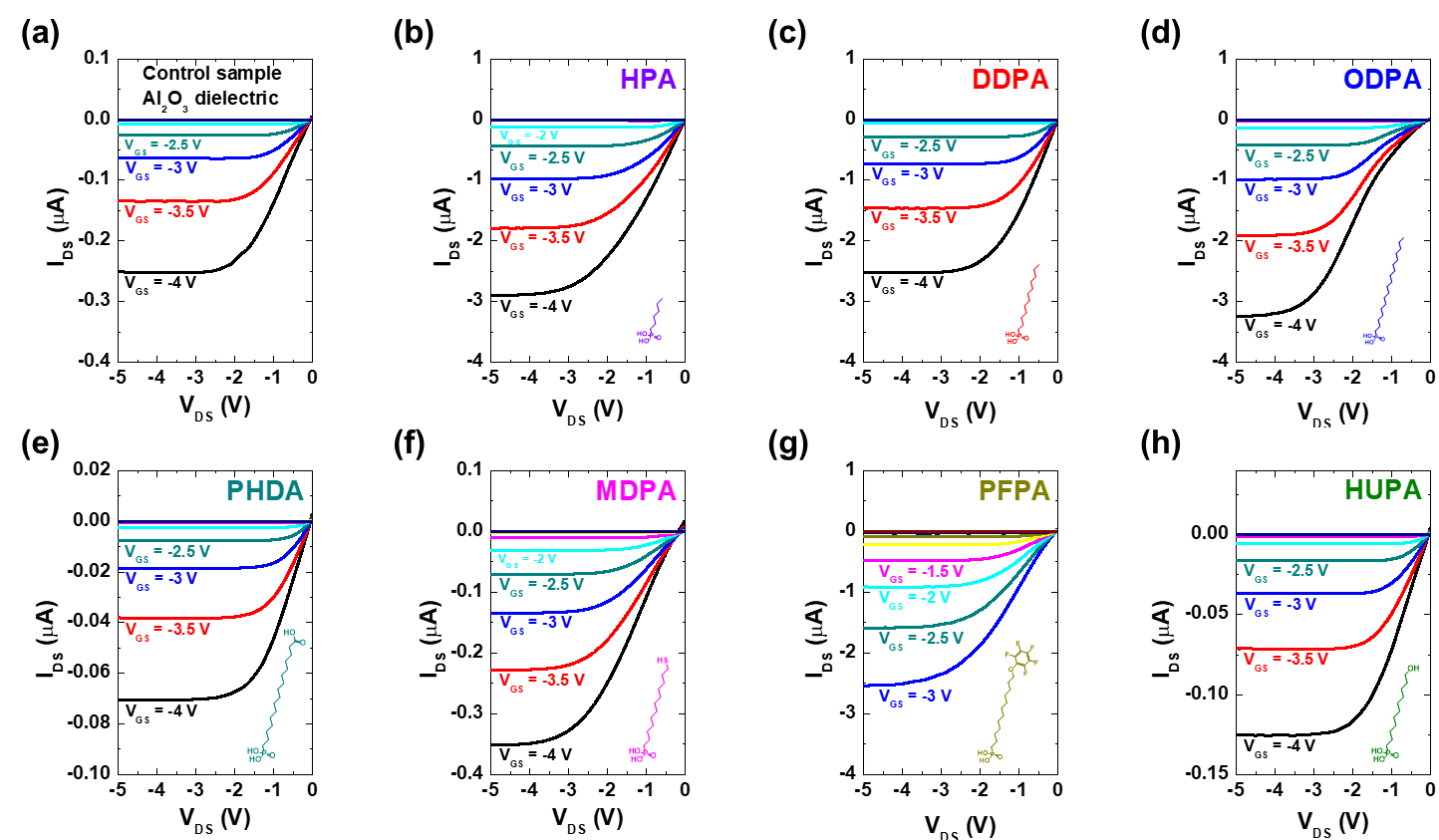
**

**Figure S2.** Output characteristics as a function of the gate voltage (*I_DS_*-*V_GS_*) of the OFET with hybrid dielectrics with various PA-SAMs (a) reference Al_2_O_3_ (b) HPA, (c) DDPA (d) ODPA, (e) PHDA, (f) MDPA, (g) PFPA and (h) HUPA.
